# Overexpression of SNHG12 regulates the viability and invasion of renal cell carcinoma cells through modulation of HIF1α

**DOI:** 10.1186/s12935-019-0782-5

**Published:** 2019-05-14

**Authors:** Qiguang Chen, Wei Zhou, Shu-qi Du, Da-xin Gong, Jun Li, Jian-bin Bi, Zhen-hua Li, Zhe Zhang, Ze-liang Li, Xian-kui Liu, Chui-ze Kong

**Affiliations:** 1 Department of Urology, The First Affiliated Hospital of China Medical University, No.155 Nanjing Street, Shenyang, 110001 Liaoning People’s Republic of China; 2 Department of Diagnostic Radiology, General Hospital of Shenyang Military Region, No.83, Wenhua Road, Shenyang, 110016 Liaoning People’s Republic of China

**Keywords:** SNHG12, MiR-199a-5p, HIF1α, Renal cancer, Long non-coding RNA

## Abstract

**Background:**

Cumulative evidences demonstrated the aberrant overexpression of Small Nucleolar RNA Host Gene 12 (SNHG12) in diverse human cancer. However, the expression status and involvement of SNHG12 in renal cell carcinoma is still elusive.

**Methods:**

The expression of SNHG12 was determined by q-PCR. The transcriptional regulation was interrogated by luciferase reporter assay. Cell viability was measured with CCK-8 kit. The anchorage-independent was evaluated by soft agar assay. Cell apoptosis was analyzed by Annexin V/7-AAD double staining. The migration and invasion were determined by trans-well assay and wound scratch closure. The in vivo tumor growth was monitored in xenograft mice model. Protein expression was quantified by immunoblotting.

**Results:**

SNHG12 was aberrantly up-regulated in renal carcinoma both in vivo and in vitro. High expression of SNHG12 associated with poor prognosis. Deficiency of SNHG12 significantly suppressed cell viability, anchorage-independent growth and induced apoptosis. In addition, SNHG12 silencing inhibited migrative and invasive in vitro and xenograft tumor growth in vivo. Mechanistically, SNHG12 modulated HIF1α expression via competing with miR-199a-5p, which consequently contributed to its oncogenic potential. MiR-199a-5p inhibition severely compromised SNHG12 silencing-elicited tumor repressive effects.

**Conclusion:**

Our data uncovered a crucial role of SNHG12-miR-199a-5p-HIF1α axis in human renal cancer.

## Background

Renal cell carcinoma (RCC) is the major form of human renal malignancies, which consists of several subtypes of cancer derived from the renal tubular epithelia [1]. In a microscopic context, there are four major histological subtypes of RCC: clear cell (conventional RCC, 75%), papillary (15%), chromophobic (5%), and collecting duct (2%) [2]. The clear cell renal cell carcinoma features richment in lipid and glycogen contents and transparency. In accordance with the Annual Cancer Statistics, approximately 340,000 new cases were diagnosed and 120,000 deaths were claimed by this disease at 2013 [3]. Besides the regular biochemical examinations for those patients with clinical manifestations and physical examination, ultrasound, computed tomography (CT) scanning and magnetic resonance imaging (MRI) are the most common diagnostic technologies for RCC [4]. Although it’s reported that more than 50% of the incidences of RCC relate to cigarette smoke, obesity and hypertension and are controllable with adjustments on lifestyle [5], the genetic aberrance in the von Hippel–Lindau (VHL) also heavily links to the etiology of RCC. VHL physiologically was identified as E3 ligase and specifically catalyzes ubiquitin attachment of hypoxia-inducible factor 1 α (HIF1α) and eventual degradation through ubiquitin–proteasome system. Compromised VHL enzymatic activity contributes to excessive transcriptional activity of HIF1α, which intrinsically dimerizes with HIF1β and translocates into nucleus to activate downstream target genes involving in diverse biological processes including neovascularization, Warburg effect and apoptosis. The sporadic mutations in mTOR and range of protein factors involving in cell epigenetics such as PBRM1, BAP1 and KDM5C have been characterized to contribute to uncontrolled cell proliferative and survival signaling in some ccRCCs as well [6]. For the early stage RCC, nephrectomy is the first choice with curative potential, whereas advanced disease required further comprehensive managements [7]. Owing to the intrinsic resistance, the clinical outcomes of conventional chemotherapy are very limited for RCC treatment [8]. Anti-angiogenesis therapies such as vascular endothelial growth factor (VEGF) and mTOR inhibitors are the mainstream targeted drugs with 30–50% objective response was recorded [9]. Nowadays, the immunotherapy targeting PD1, PD-L1 and CTL4 received increasingly interests [10]. Long non-coding RNAs (lncRNA) are defined as RNA molecules longer than 200 bp lack of protein-coding potential, which account for approximately 80% of human transcripts [11]. Around 300 lncRNAs were archived and curated in LncRNAdb database (http://www.lncrnadb.org) as of July 2017. The kaleidoscope of biological roles of lncRNAs have been progressively uncovered involving in complex gene regulation network [12]. It’s been accumulatively characterized that lncRNAs target multiple transcription components either in cis- or trans-manner in eukaryotes, ranging from transcription activators, repressors, RNA polymerase II to cDNA duplex [13]. Additionally, lncRNAs also involve in multiple stages during mRNA maturation processing across splicing, translocation, translation and decay in a manner reminiscent of the mode of action of microRNAs and snoRNAs [14]. The importance of lncRNAs in chromatin epigenetics have been unraveled as well, which mediate imprinting, sexual chromosome silencing and telomere stabilization [15]. Small nucleolar RNA host gene 12 (SNHG12) has been increasingly recognized involving in variety of human cancers such as human osteosarcoma cell, nasopharyngeal carcinoma cell, and human endometrial carcinoma. SNHG12 was first characterized over-expressing in osteosarcoma cells [16], which promoted cell proliferation and migration by up-regulating angiomotin gene expression. Zhang et al. have identified significant up-regulation of SNHG12 in brain microvascular endothelium after cerebral ischemia, which might play potential pathological roles in mediating endothelial response to ischemic stimuli [17]. In colorectal cancer cells, Wang et al. demonstrated that long non-coding SNHG12 promoted cell growth and inhibited cell apoptosis via boosting cell cycle progression [18]. While in triple-negative breast cancer, Wang et al. showed c-Myc-induced up-regulation of SNHG12 regulated cell proliferation, apoptosis and migration likely through MMP13 signaling [19]. Zhu et al. revealed the potential biomarker role of SNHG12 for diagnosis and treatment purpose in lung adenocarcinoma [20]. Wang et al. reported knockdown of SNHG12 inhibited cell growth and induced apoptosis by up-regulating miR-138 in non-small cell lung cancer [21]. Overexpression of SNHG12 also contributed multidrug resistance through activating the MAPK/Slug pathway by sponging miR-181a in non-small lung cancer [22]. Zhang et al. also revealed the regulatory effect of SNHG12 in gastric cancer progression by acting as a molecular sponge of miR-320 [23]. The microRNA sponging function of SNHG12 also has been disclosed in osteosarcoma, wherein promoted tumorigenesis and metastasis by up-regulating Notch2 via competing with miR-195-5p [16]. However, the expression status and potential involvement of SNHG12 in renal cancer has not been fully understood. Here we attempted to analyze the relative expression of SNHG12 and address its mechanistic relation to renal cancer.

## Materials and methods

### Clinical samples

The procedure involving human-related investigation was pre-approved and authorized by the Ethics Committee of The First Affiliated Hospital of China Medical University. In total, 20 renal carcinoma and adjacent benign tissues were obtained from patients who were enrolled in this study from The First Affiliated Hospital of China Medical University and informed with written consents (age 57.5 ± 9.3; male 75%). The diagnoses of renal carcinoma were validated by three experienced clinical pathologists. The fresh tissue samples were restored in liquid N_2_ for future analysis.

### Cell culture

The 293T cell, renal epithelial HK-2 cell and kidney carcinoma A498, 786-0, 769-p, Caki-1, Caki-2 and ACHN were ordered and authenticated by the America Typical Culture Collection (ATCC, VA, USA). 293T cells were cultured in high-glucose DMEM medium (Gibco, CA, USA) with 10% FBS (Hyclone, MA, USA) and 1% penicillin/streptomycin (Gibco, CA, USA). All the other cells were maintained in RPMI-1640 medium (Gibco, CA, USA) supplemented with 10% fetal bovine serum and 1% P–S–G. The exponential cells were cultured in 37 °C humidified incubator with 5% CO_2_.

### Transfection

The indicated plasmids were transfected into either Caki-1 and ACHN cells using Lipofectamine 2000 (Invitrogen, CA, USA) following the provider’s manual. Briefly, the exponential Caki-1 or ACHN cells were cultured in 6-well plate the day for 24 h. The shSNHG12: SNHG12-specific shRNA; shNT: negative control shRNA; pmiR-199a-5p: miR-199a-5p expressing plasmid; pCtrl: empty vector; SNHG12: SNHG12-expressing plasmid; anti-miR-199a-5p: miR-199a-5p antagomir were packaged with Lipofectamine 2000 and homogenously added into each well. The efficiency in cell transfection was assured with parallel GFP assay. The shSNHG12 and the miR-199a-5p mimic were purchased from GenePharma (Suzhou, China). The sequences were provided as following:

- shSNHG12-sense: 5′-CACCGAGGGAGACCAGAAGATAATGTTCAAGAGACATTATCTTCTGGTCTCCCTCTTTTTTG-3′;
- ShNC-sense: 5′-CACCGTTCTCCGAACGTGTCACGTCAAGAGATTACGTGACACGTTCGGAGAATTTTTTG-3′;
- miR-199a-5p sense: 5′-CCCAGUGUUCAGACUACCUGUUC-3′;
- miR-NC sense: 5′-UUCUCCGAACGUGUCACGUTT-3′.

### Real-time PCR

The cellular RNA was isolated from indicated samples or cells by Trizol (Invitrogen, CA, USA) following the manufacturer’s instruction. The concentration was measured with Nanodrop 1000 (ThermoFisher, MO, USA). The integrity of RNA was quality-checked by BioAnalyzer 2100 (Agilent, CA, USA). cDNA was prepared from each 1 μg of RNA via reverse transcription reaction using High-Capacity cDNA Synthesis Kit (ThermoFisher, MO, USA) according to the provider’s instructions. The primer sequences were provided in Table 1.

**Table 1:** RT-PCR primers and shRNAs oligonucleotides

*Primers used for RT-PCR*	
SNHG12-F	TCTGGTGATCGAGGACTTCC
SNHG12-R	ACCTCCTCAGTATCACACACT
GAPDH-F	GCTCTCTGCTCCTCCTGTTC
GAPDH-R	ACGACCAAATCCGTTGACTC
*shRNAs/shRNA oligonucleotides*	
Scrambled	GGAATCTCATTCGATGCATAC
shSNHG12	TGCACTAGCTGGCATCACCGC

### Dual-luciferase reporter assay

To interrogate the regulation of miR-199a-5p on expression of SNHG12, the full-length SNHG12 cDNA was amplified using the following PCR primers and sub-cloned into pmirGLO Dual-Luciferase miRNA Target Expression Vector (Promega, MI, USA) via double restriction digestion (*Xba*I and *Nhe*I, New England Biolabs, MA, USA) and T4 ligation. The scrambled mutant was generated by mutagenesis PCR.

- SNHG12-forward: 5′-CCTTTCTCCCCGCCGCATTC-3′;
- SNHG12-reverse: 5′-ATTGATCACCTCTGAAGTTC-3′;
- SNHG12-mut forward: 5′-TCCCATCGGATCTCTTGTGTGGTCTTGGTGGT-3′;
- SNHG12-mut reverse: 5′-ACCACCAAGACCACACAAGAGATCCGATGGGA-3′.

And co-transfected Caki-1 or ACHN cells with miR-199a-5p. Cells were harvested 48 h later and relative luciferase activities were measured with Dual Luciferase Assay System (Promega, WI, USA).

### Western blotting

The cell lysates were prepared in RIPA lysis buffer. Equal amount of protein was subjected to SDS-PAGE resolution and transferred onto PVDF membrane on ice. The protein blots were hybridized with indicated primary antibodies immediately after blocking with 5% milk at 4 °C overnight. The second round of incubation with secondary antibody was performed at room temperature for 1 h. The target protein was visualized with the enhanced chemiluminescence method (ECL, Millipore, MO, USA).

### Cell viability assay

The relative cell viability was determined by the Cell Counting Kit-8 (CCK-8 Kit, Dojindo, Dalian, China) according to the provider’s manual. 10,000 cells were plated into each well of 96-well plate in triplicate for 72 h. 10 µL of CCK-8 solution was subsequently applied into each well and allowed for reaction at 37 °C for 1 h. The absorption at 450 nm was recorded by microplate reader (Xmark, Bio-Rad, CA, USA) and relative cell count was calculated.

### Soft agar assay

The colony formation capacity was determined by soft agar assay. Caki-1 and ACHN were transfected with either negative control (shNT) or SNHG12-specific shRNA (shSNHG12) and allowed by puromycin (Sigma, MO, USA) selection. The success in establishment of stable cell line was confirmed by real-time PCR. The single-cell suspension then was prepared via trypsin digestion in 2× RPMI modified medium. The cell concentration was calculated with hemocytometer and adjusted to 10^4^ cells/500 μL. The equal volume of cell solution and 1% low-melting point agarose was mixed and laid on the top of precast 1.2% agarose cushion. The formed colonies after 2 weeks were stained with 0.05% crystal violet and counted under light microscope.

### Cell apoptosis assay

The cell apoptosis was determined by Annexin V/PI staining method. Briefly, the cells were transfected as described above, and 24 h later the exponential cells were harvested with trypsin and resuspended in HEPES buffer. Double-staining with Annexin V and 7-AAD (Sigma, MO, USA) was performed at room temperature for 20 min free-of-light. The apoptotic cells were analyzed by flow cytometry (BD Biosciences, CA, USA).

### Transwell assay

Both cell migration and invasion experiments were conducted with Transwell polycarbonate membrane cell culture inserts (BD Biosciences, CA, USA). The cells were pre-starved in serum-free medium, then single-cell suspension was prepared via trypsin digestion. The cell concentration was calculated and adjusted to 1 × 10^5^/100 μL. Each 100 μL cell solution was poured onto the upper compartment of polycarbonate Transwell chamber (Matrigel was applied for the filter for invasion assay (BD Biosciences, CA, USA)). 750 μL of complete culture medium containing 10% FBS was filled in the lower chamber as attractant. After 20 h, the free cells inside insert were completely wiped off with cotton Q-tips. The migrated/invaded cells were fixed with 4% paraformaldehyde and followed by crystal violet staining for 15 min. The cells were counted in three independent fields under optical microscope to calculate the transwell capacity.

### Scratch healing assay

Scratch healing assay was employed to evaluate the cell migrative behavior. Either SNHG12-deficient or -proficient Caki-1 and ACHN cells were seeded into 6-well plate. The wound was introduced into each well using sterile pipette tips to draw a straight line. The wound-closing was regularly recorded and measured under light microscope.

### Xenograft tumor

To investigate the potential impact on tumor progression in vivo, we established stable SNHG12-deficient cell line in Caki-1 via puromycin selection (Sigma, MO, USA). Totally, twenty 4-week old female BALB/c-nude mice (20–22 g) were purchased from the National Laboratory Animal Center (NLAC), and equally assigned into shNT and shSNHG12 recipient groups after 1-week quarantine. All animal related experiments were performed in a SPF-class environment and the protocol was pre-approved by the Committee of Animal Care and Use of The First Affiliated Hospital of China Medical University. The animals were housed at 20–22 °C and 40–60% humidity with a 12-h light/dark cycle. Standard animal diet and water were freely available. The single-cell resuspension was prepared from log-phase cells and mixed with equal volume of Basal Membrane Extract (BD BioSciences, CA, USA). The mixture was subcutaneously injected into the lower flank of immunodeficient mice. Tumor progression was observed twice a week and volume were calculated. The animals were sacrificed at day 35 post-inoculation and xenograft tumor was resected and weighed.

### FISH assay

RNA hybridization was applied to determined the intracellular localization of SNHG12 with Empire Genomics SNHG12 FISH probe (SNHG12-20-OR and GAPDH-20-OR, Empire Genomics, NY, USA) following the manufacturer’s recommendations. The nuclear was counter-stained with Hoechst 33342. The representative images were captured on Zeiss LSM 720 confocal microscope.

### Statistical analysis

All results shown in this study was physically repeated at three times unless indicated. Data processing was facilitated by GraphPad PRISM 6.0 software. Statistical comparison was performed using ANOVA followed by Turkey’s test. The p value was calculated and p < 0.05 was considered as significantly different.

## Results

### Over-expression of SNHG12 in human renal tumor

**Fig. 1:**
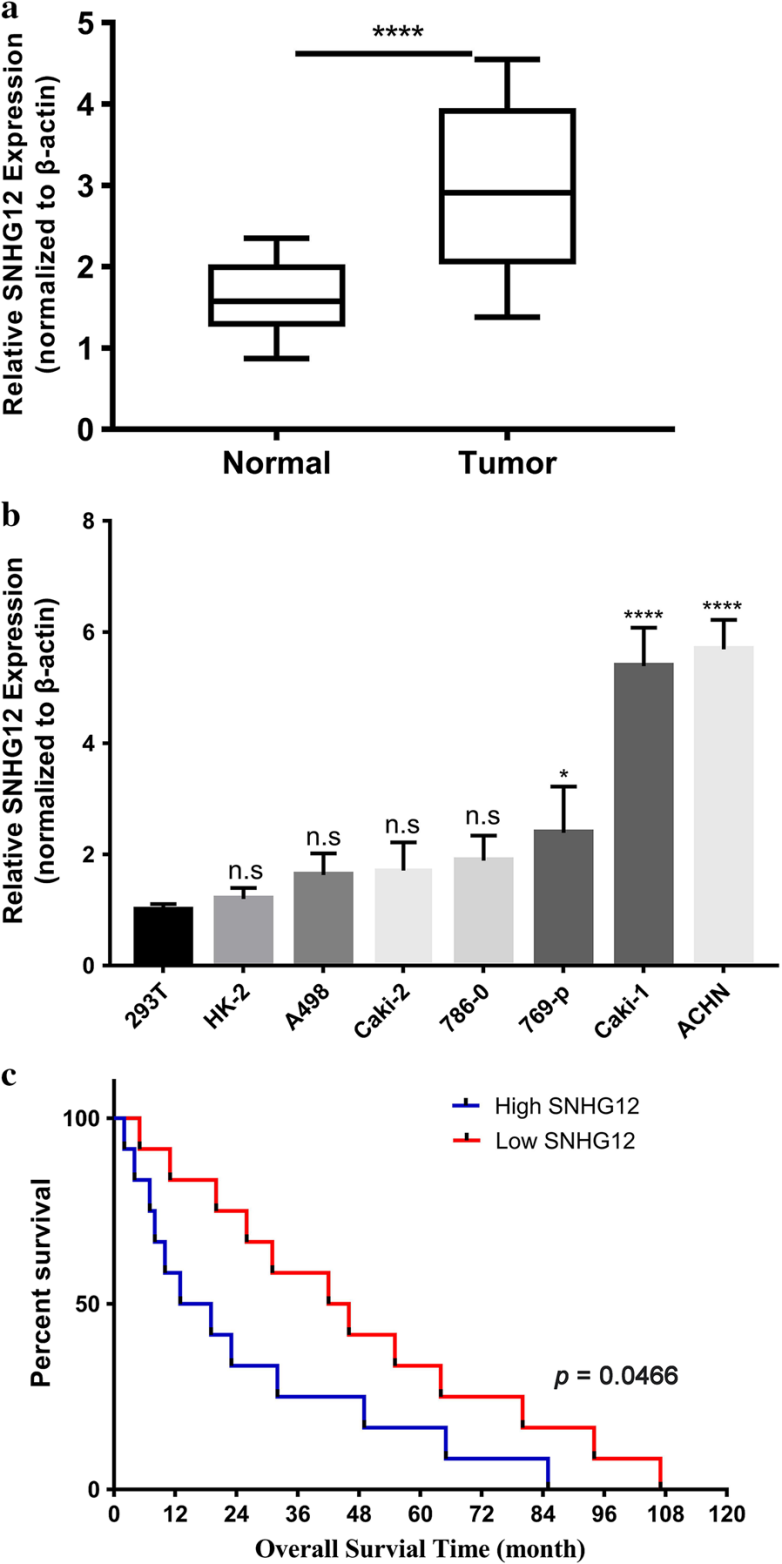
Overexpression of SNHG12 in human renal cancer. **a** The expression of SNHG12 was determined by real-time PCR and normalized to β-actin in human renal cancer samples (n = 20 pairs); **b** Relative expression of SNHG12 was measured by real-time PCR in human renal cancer cell line panel (n = 7) in comparison with both 293T cell and renal epithelial cell line HK-2. Ns, no significance, *p < 0.05, ****p < 0.0001; **c** Kaplan–Meier curve of cumulative survival in renal cancer patients with high SNHG12 (n = 10) and low SNHG12 expression (n = 10)

The relative expression of SNHG12 in renal carcinoma was determined first by real-time PCR in 20 pairs of tumor and adjacent benign tissue. As shown in Fig. 1a, SNHG12 was significantly higher in most of renal tumors in comparison with normal control, which indicated the potential oncogenic activity of SNHG12. We further quantified SNHG12 expression in our available renal cancer cell lines regardless of the mutation status of VHL. In line with the in vivo results from cancer patients, SNHG12 mRNAs were up-regulated in all of subject cancer cell lines than in 293T cells and renal epithelial HK-2 cells (Fig. 1b). The Caki-1 and ACHN possessed the highest level of SNHG12 among all tested cell lines, which were selected for the further investigation. In agreement with several previous studies into solid tumors, our data acquired both in vivo and in vitro showed the universal overexpression of SNHG12 in renal carcinoma. Moreover, the Kaplan–Meier analysis of overall survival in our clinical data exhibited more favorable prognosis in patients with low level of SNHG12 (48 months in low-SNHG12 group vs. 24 months in high-SNHG12 group, Fig. 1c), which consolidated the oncogenic role of SNHG12 in this disease.

### SNHG12-knockdown inhibited cell viability, anchorage-independent colony formation and induced apoptosis in renal cancer cells

**Fig. 2:**
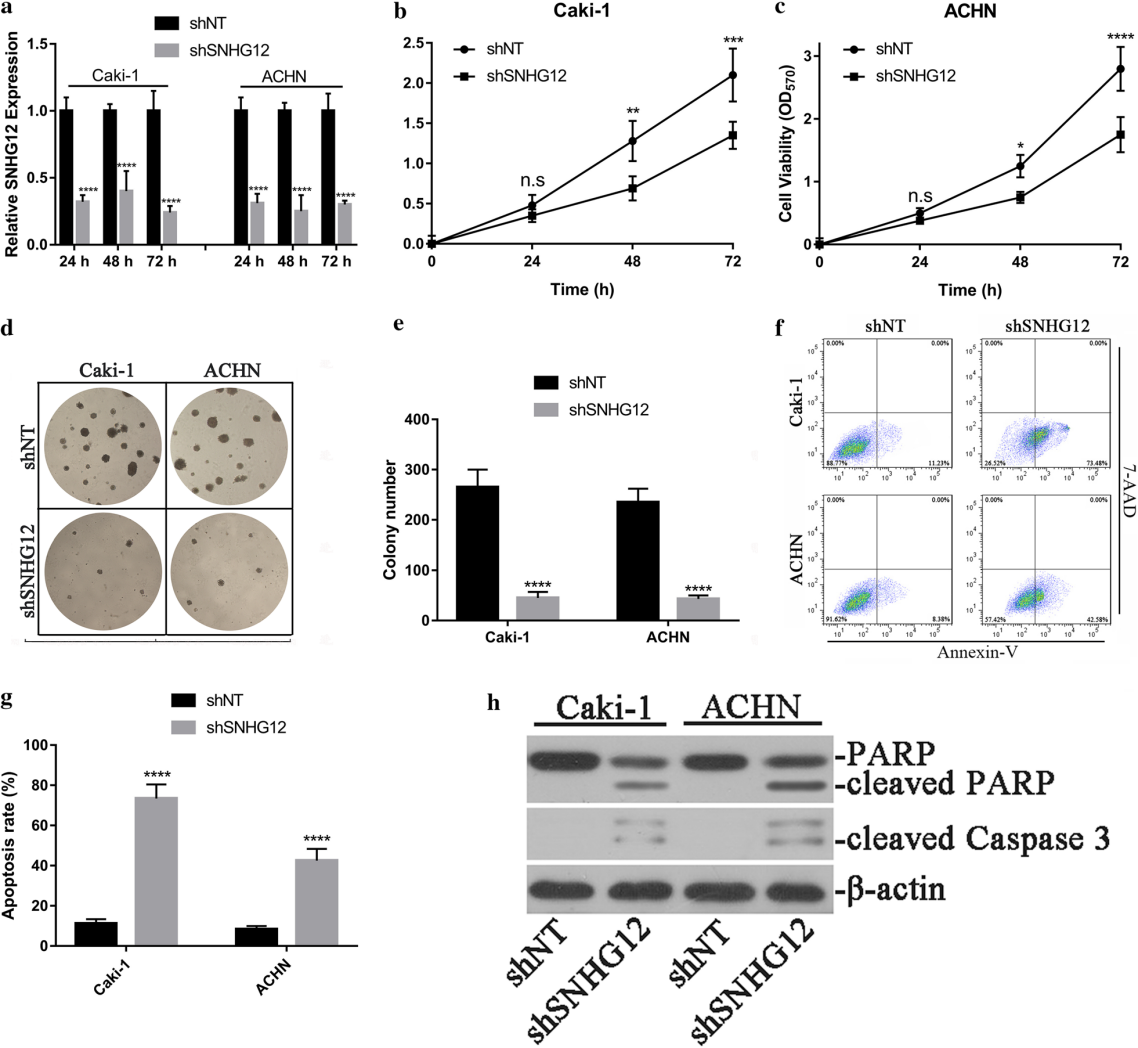
SNHG12-knockdown inhibited malignant progression in renal cancer cells. **a** Caki-1 and ACHN cells were transfected with either scramble or SNHG12 shRNA using lipofectamine 2000. The knockdown was measured by real-time PCR at 24, 48 and 72 h post-transfection. ****p < 0.0001; **b**, **c** Cell viability was determined in SNHG12-deficient ACHN and Caki-1 cells with CCK-8 kit. **d** The anchorage-independent growth of both SNHG12-proficient and -deficient ACHN and Caki-1 cells was interrogated by soft agar assay in triplicate, the statistical results from three individual fields were shown in pane E, ****p < 0.0001. **f**, **g** The apoptotic cells were analyzed with Annexin-V/7-AAD double-staining method, followed by flow cytometry semi-quantitation. ****p < 0.0001. **h** Cleaved PARP and Caspase 3 in response to SNHG12 silencing were determined by western blotting. β-Actin was used for loading control

Our previous results demonstrated that over-expression of SNHG12 was prevalent in renal carcinoma, which intimately associated with poor outcomes. Then we attempted to experimentally validate the oncogenic properties of SNHG12 in renal cancer cell lines. Caki-1 and ACHN cells were chosen for this purpose owing to the high abundance of SNHG12. The shRNA-mediated knockdown of SNHG12 was first confirmed by quantitative PCR at 24, 48 and 72 h post-transfection, more than 50% reduction was achieved in both Caki-1 and ACHN cells during our experiment window (Fig. 2a). SNHG12 deficiency tremendously inhibited cell viability in Caki-1 (Fig. 2b) and ACHN cells (Fig. 2c). In addition, the colony formation capacity was remarkably compromised in stable SNHG12-knockdown cells in comparison to the wild-type counterparts (Fig. 2d, e). Furthermore, SNHG12-deficiency induced spontaneous apoptosis in both cell lines (72.3 ± 8.1% vs. 11.3 ± 0.9% in Caki-1, 42.3 ± 7.4% vs. 8.0 ± 0.5% in ACHN, Fig. 2f, g). The cleaved PARP and Caspase 3 were dramatically induced in SNHG12-deficient cells as well (Fig. 2h). Our results clearly showed that impairment of SNHG12 significantly inhibited viability, anchorage-independent growth and induced cell apoptosis in renal carcinoma cell lines.

### SNHG12-deficiency repressed migration and invasion in renal cancer cells

**Fig. 3:**
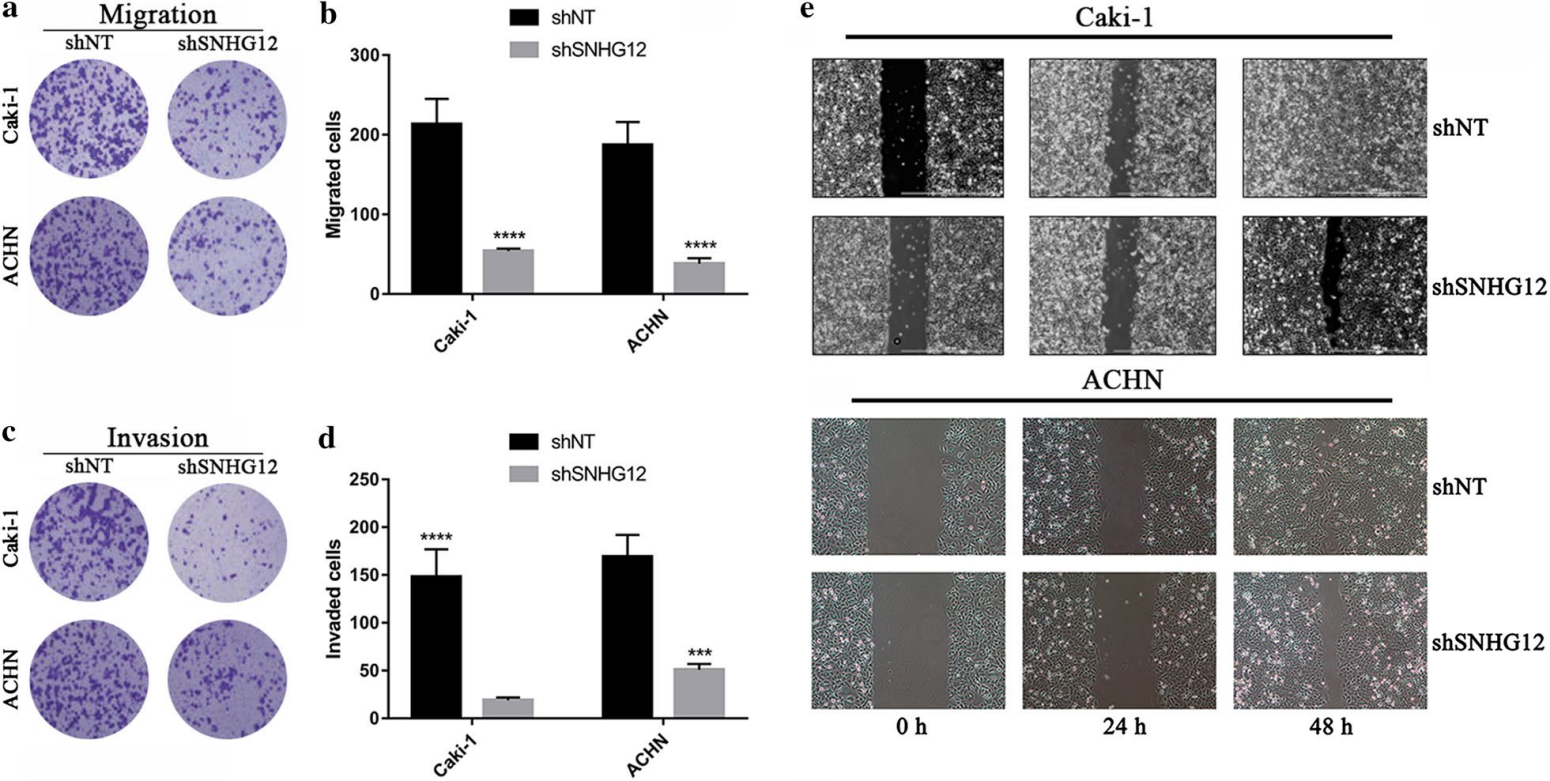
SNHG12-silencing suppressed migration and invasion of renal cancer cells. **a**, **b** The migratory capacity was evaluated using transwell chamber. The representative images were shown in **a** and the statistical results in **b**. ****p < 0.0001. **c**, **d** The invasive capacity of indicated cells were determined in Matrigel-coated transwell assay. The representative images were shown in **c** and the statistical results in **d**. ***p < 0.001, ****p < 0.0001. **e** Scratch closure assay was performed to assess migration in SNHG12-deficient Caki-1 (top pane) and ACHN cells (bottom pane). The wound closure rate was monitored at 0, 24 and 48 h respectively

The previous results provided evidences that SNHG12 knockdown in renal cancer cells significantly suppressed malignant growth. Next, we sought to further investigate the potential impact of SNHG12 on migration and invasion. The migrative behavior was evaluated by the transwell chamber, which demonstrated that SNHG12-deficiency markedly repressed the migratory capacity of Caki-1 and ACHN (Fig. 3a, b). Likewise, in the Matrigel-coated transwell chamber, the number of invaded cells was significantly decreased by SNHG12 knockdown (Fig. 3c, d). The wound closure experiment was conducted to validate the compromised migrative capacity in SNHG12-deficient renal carcinoma cells as well. Consistent with results from transwell assay, the wound healing speed was decelerated in SNHG12-deficient cells compared to the proficient ones (Fig. 3e), which highlighted the critical role of SNHG12 in promotion of renal cancer cell migration. In summary, our data provided evidences that SNHG12 played important roles in promotion of renal cancer metastasis during disease progression.

### SNHG12-deficiency inhibited xenograft tumor progression

**Fig. 4:**
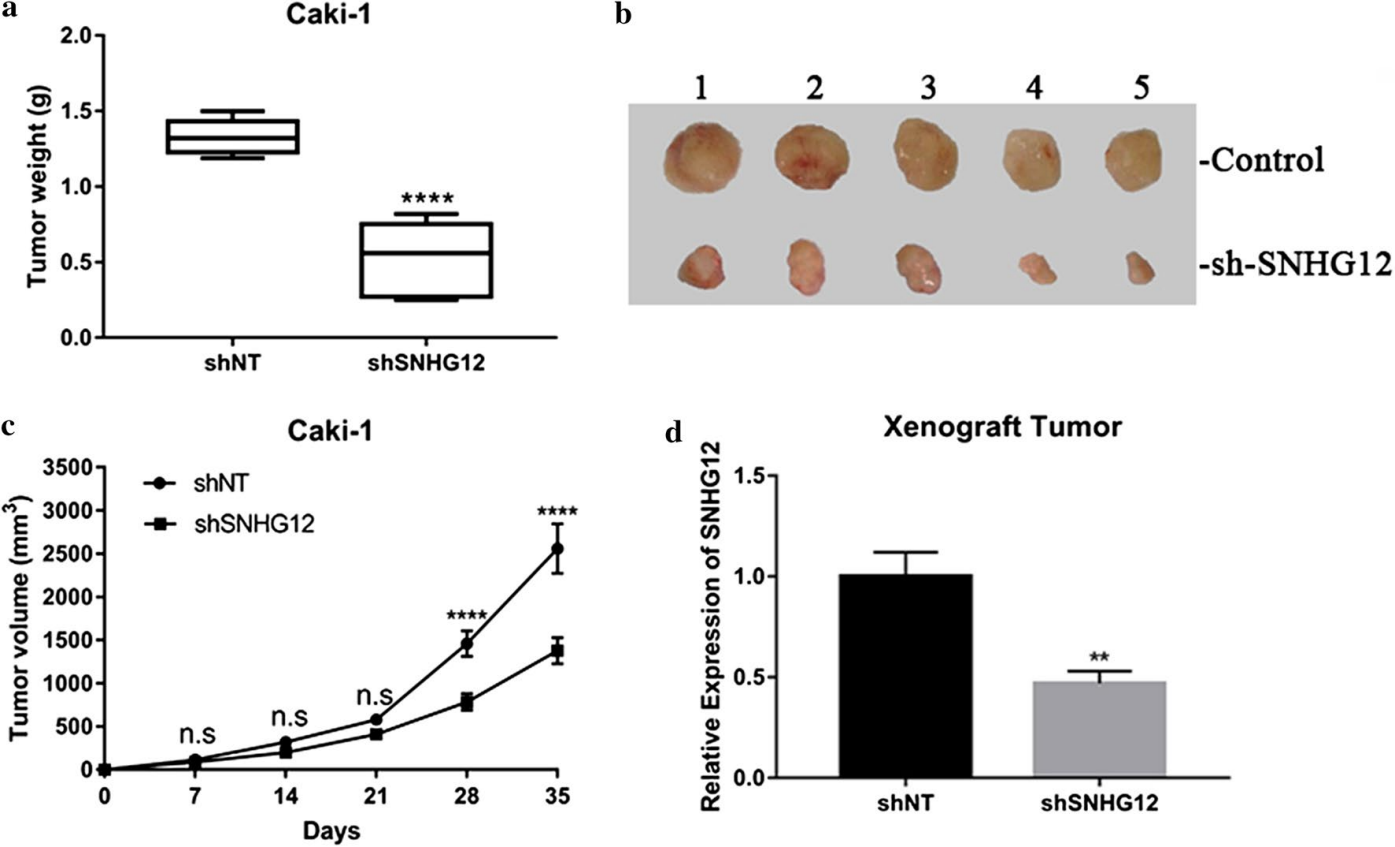
SNHG12 deficiency suppressed xenograft tumor growth in vivo. **a** The stable SNHG12-deficient Caki-1 cell line was established and subcutaneously inoculated into nude mice. The xenograft tumors were weighed after sacrifice, ****p < 0.0001. **b** Macroscopic images of representative xenograft tumors from both control and shSNHG12 mice resected at week 5 post-inoculation. **c** The tumor growth was monitored by digital caliper at day 0, 7, 14, 21, 28 and 35 post-injection respectively and the volume was calculated with the formula: volume = (width)^2^ × length/2. Ns, no significance, ****p < 0.0001. **d** The persistent silencing of SNHG12 in xenograft tumor was confirmed by real-time PCR. **p < 0.01

Next, we sought to consolidate the oncogenic properties of SNHG12 observed in cell culture in vivo via employment of xenograft mice mode. Caki-1 cells persistently expressing either scramble or shSNHG12 were subcutaneously implanted into lower flanks in immunodeficient mice, which were sacrificed while the maximum xenograft tumor approaching 3000 mm^3^. The xenograft tumor progression was significantly inhibited by SNHG12 knockdown, with the average weight of tumor was 0.53 ± 0.09 g in SNHG12-deficient recipients versus 1.33 ± 0.05 g in control group (Fig. 4a). The representative images of xenograft tumor resected from subject animals at the endpoint of experiment was showed in Fig. 4b with much smaller size in SNHG12-knockdown mice. The great delay was observed in respect to tumor sized was observed in SNHG12-deficient mice in comparison with SNHG12-proficient counterparts (Fig. 4c). The persistent suppression of SNHG12 expression in recipient mice was verified by real-time PCR in the sacrificed mice (Fig. 4d). Here we consolidated the phenotype in renal carcinoma xenograft tumor and validated the oncogenic activity of SNHG12 in vivo.

### SNHG12 regulated HIF1α via endogenous competition with miR-199a-5p

**Fig. 5:**
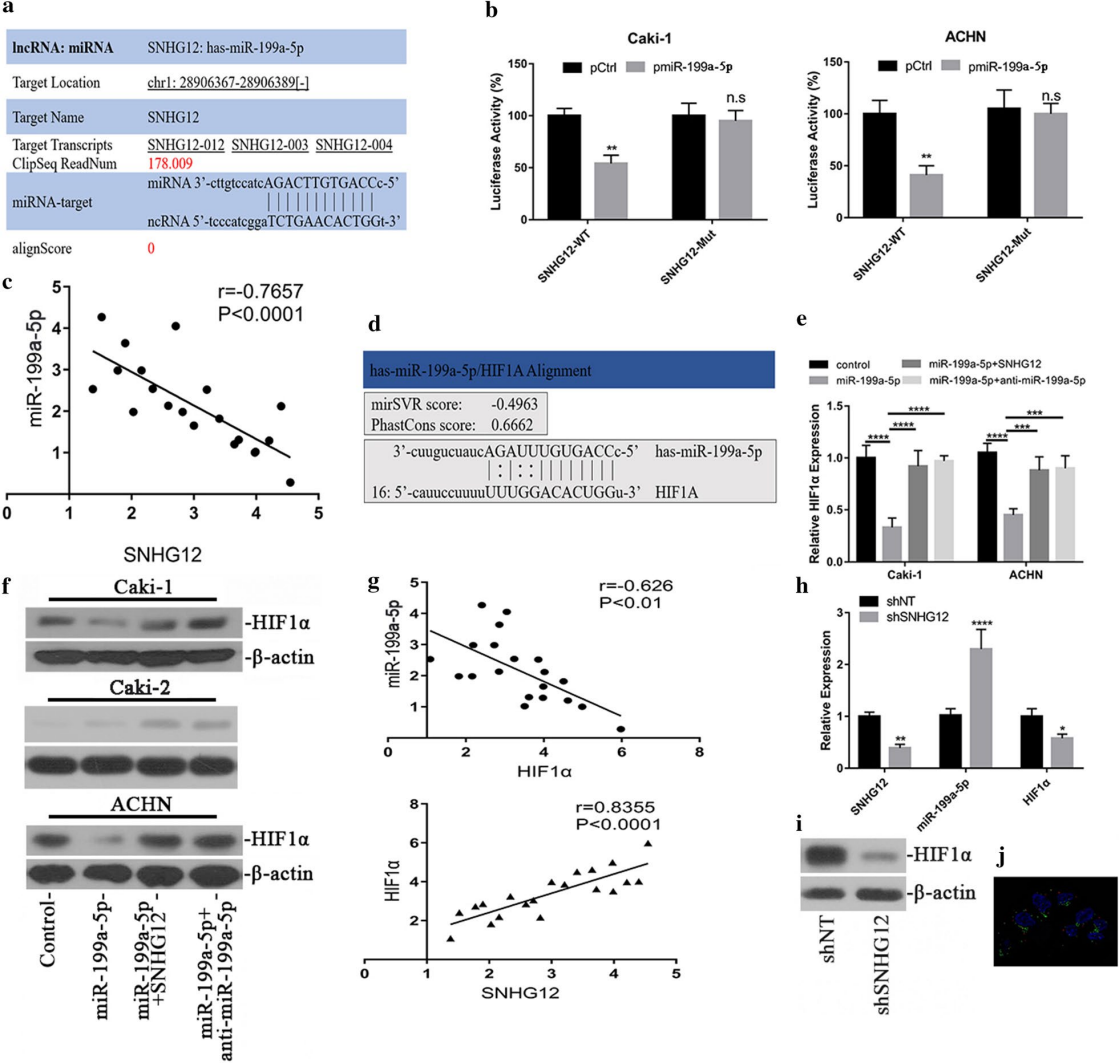
SNHG12 regulated HIF1α via endogenous competition with miR-199a-5p. **a** Alignment between miR-199a-5p seed region and SNHG12 transcript using Starbase online tool. **b** Luciferase reporter assay was performed to validate the regulatory effect of miR-199a-5p on SNHG12 transcript. Either wild-type or putative binding site mutant SNHG12 was fused to luciferase, which was co-transfected with miR-199a-5p into ACHN and Caki-1 cells. The relative luciferase activities were measured with the Bright-Glo Luciferase Assay System **p < 0.01, ns, no significance. **c** Correlation between miR-199a-5p and SNHG12 transcripts in renal cancer samples (n = 20). **d** Alignment between miR-199a-5p and putative target sites in HIF1α 3′UTR by microRNA online tool. **e** miR-199a-5p negatively modulated HIF1α expression, which was antagonized by SNHG12. Exogenous scramble sequence, miR-199a-5p, SNHG12 or anti-miR-199a-5p were transfected into Caki-1 (left) and ACHN (right) cells in combination as indicated, the relative expression of HIF1α was measured by real-time PCR in indicated cells. **f** The HIF1α protein was quantified by immunoblotting. β-Actin served as loading control. ***p < 0.001, ****p < 0.0001. **g** Correlation between miR-199a-5p and HIF1α (upper pane, r = − 0.626, p < 0.01), SNHG12 and HIF1α (lower pane, r = 0.8355, p < 0.0001) in renal cancer samples (n = 20). SNHG12-miR-199a-5p-HIF1α axis was analyzed by q-PCR (**h**) and western blotting (**i**) in xenograft tumor. *p < 0.05, **p < 0.01, ***p < 0.001. **j** Subcellular localization of SNHG12 was analyzed RNA hybridization with the specific Stellaris RNA FISH probes followed by confocal microscope imaging. SNHG12 was detected in red channel, while cytoplasmic GAPDH transcript was detected in green channel. The nuclei were counter-stained with DAPI

There were emerging evidences suggested the endogenous competing function of long non-coding RNA with miRNAs [24]. Here we sought to examine this possibility in respect to oncogenic feature of SNHG12 following this direction. With aid of lncRNABase online algorithm (http://starbase.sysu.edu.cn/starbase2/mirLncRNA.php), we first predicted the putative miR with the possibility to directly associate with SNHG12 and identified miR-199a-5p on the top list of candidates. The alignment of SNHG12 and miR-199a-5p was illustrated in Fig. 5a. The direct regulation of SNHG12 by miR-199a-5p was interrogated in our luciferase reporter assay and relative expression of SNHG12 in our system was interrogated with luciferase activity. The ectopic expression of miR-199a-5p led to about 45% reduction of SNHG12 transcript in Caki-1 and 60% reduction in ACHN cells (Fig. 5b) respectively. The putative miR-199a-5p recognizing sites in SNHG12 transcript was further experimentally validated. As shown in Fig. 5b, mutation introduced into the suspected region of SNHG12 completely abolished miR-199a-5p-conferred inhibition on luciferase activities. The relationship between relative expression of miR-199a-5p and SNHG12 was analyzed in renal carcinoma samples, and the inverse correlation has been plotted in Fig. 5c (R^2^ = 0.5864, p < 0.0001). Our data indicated that SNHG12 might involve in gene regulatory network of miR-199a-5p via molecularly sponging miR-199a-5p.

Hypoxia response is fundamental to tumorigenesis in renal carcinoma [25]. The previous study by Jiang et al. showed that miR-199a-5p involved in regulation of HIF1α in human epilepsy [26], which prompted us to clarify this possibility in renal carcinoma. With aid of bioinformatics tools from microRNA.org, we certainly identified the putative target site of miR-199a-5p in 3′UTR region of HIF1α, and alignment between miR-199a-5p and HIF1α was illustrated in Fig. 5d. Ectopic expression of miR-199a-5p remarkably decreased endogenous HIF1α at transcript level by Q-PCR in both Caki-1 and ACHN cells (Fig. 5e). This phenotype was further confirmed at protein level as shown in Fig. 5f. The predominance of miR-199a-5p in this inhibitory action was confirmed by employment of miR-199a-5p-specific inhibitor simultaneously, which readily abolished this phenotype (Fig. 5e, f). In VHL-proficient Caki-2 cells, the low level of HIF1α was slightly stimulated by both SNHG12 and miR-199a-5p inhibitor. Notably, the negative correlation between miR-199a-5p and HIF1α and positive correlation between SNHG12 and HIF1α have been characterized in renal cancer patients (Fig. 5g). The expression status of SNHG12-miR-199a-5p-HIF1α axis was also analyzed in xenograft tumor tissue at mRNA and protein level (Fig. 5h, i), which was consistent with our previous results acquired from cell culture. In support of its mode-of-action as endogenous competing RNA, we further demonstrated the major cellular localization of SNHG12 in cytoplasm using FISH probe (Fig. 5j).

### miR-199a-5p antagonism abolished SNHG12-deficiency rendered tumor suppressive effects

**Fig. 6:**
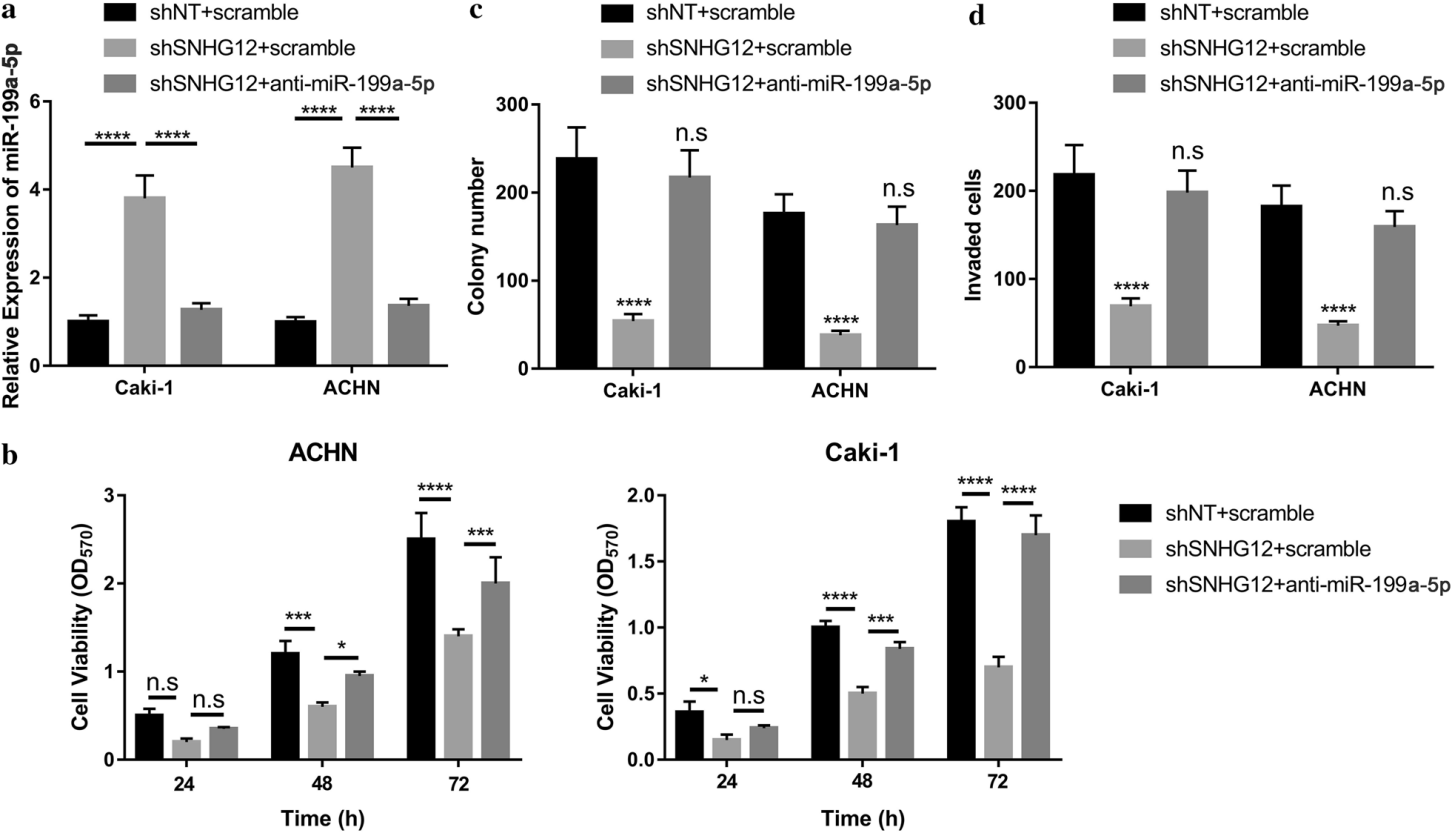
miR-199a-5p-inhibition abrogated SNHG12-silencing elicited tumor suppressive effect. **a** The relative miR-199a-5p level in response to SNHG12 knockdown or specific inhibitor was determined by real-time PCR in both Caki-1 and ACHN cells. ****p < 0.0001. **b** The suppressive effect on cell viability of SNHG12 deficiency was relieved by miR-199a-5p inhibition. The cell viability was monitor at 0, 24, 48 and 72 h respectively using CCK-8 kit. ns, no significance, *p < 0.05, ***p < 0.001, ****p < 0.0001. **c** Colony formation assay was performed to measure the anchorage-independent growth of indicated cells. ns, no significance, ****p < 0.0001. **d** The invasive capacity was determined by Matrigel-coated Transwell assay. ns, no significance, ****p < 0.0001

We previously proposed that SNHG12 might act as competing endogenous RNA (ceRNA) to associate with miR-199a-5p in fine-tune HIF1α expression. Then, we sought to evaluate the oncogenic properties of SNHG12 in renal cancer along this direction. The intrinsic miR-199a-5p in SNHG12-deficient Caki-1 and ACHN cells was examined by real-time PCR for this purpose. As shown in Fig. 6a, SNHG12-knockdown induced tremendous up-regulation of miR-199a-5p, which was abolished by miR-199a-5p antagonist. MiR-199a-5p antagonism almost abolished the inhibitory effect on cell viability elicited by shSNHG12 (Fig. 6b). Likewise, the compromised colony formation capacity in SNHG12-deficient cells was readily restored by miR-199a-5p-specific inhibitor (Fig. 6c). MiR-199a-5p antagonist stimulated transwell invasion in the SNHG12-silencing cells as well (Fig. 6d). Our data clearly elucidated that miR-199a-5p predominately antagonized the oncogenic feature of SNHG12 in renal cancer.

## Discussion

SNHG12 is a novel lncRNA with increasingly recognized potential involvements in diversity of human malignancies. Notably, Lan et al. demonstrated that SNHG12 promoted tumorigenesis and metastasis by targeting miR-199a/b-5p in hepatocellular carcinoma [27]. Consistently, in this study, we first characterized aberrant overexpression of SNHG12 in renal carcinoma both in vivo and in vitro. High expression of SNHG12 significantly associated with poor prognosis in renal cancer patients. SNHG12 knockdown remarkably inhibited cell viability, induced anoikis and stimulated cell apoptosis. Moreover, SNHG12-deficiency compromised both migrative and invasive capacity in renal cancer in vitro as well. The in vitro phenotype was further consolidated in vivo by employment of xenograft tumor mice model. Mechanistically, we elucidated that SNHG12 functioned as competing endogenous lncRNA against miR-199a-5p, and SNHG12 inversely correlated to endogenous miR-199a-5p in renal carcinomas. Noteworthily, we unraveled that HIF1α as one of the target genes of miR-199a-5p, whereas the inhibitory action was antagonized by SNHG12. The predominant role of miR-199a-5p involving in the oncogenic signaling of SNHG12 was consolidated by introducing miR-199a-5p-specific inhibitor, wherein the anti-tumor effect elicited by SNHG12-knockdown was completely abrogated. Our study for the first time highlighted the critical role of SNHG12-miR-199a-5p-HIF1α axis underlying the malignant features of renal carcinoma.

The hypoxia response is the most critical to incidence of renal carcinoma, which is frequently associated with mutations of Von Hippel–Lindau (VHL) gene. VHL physiologically was identified as E3 ligase and specifically catalyzes ubiquitin attachment of hypoxia-inducible factor 1 α (HIF1α) and eventual degradation through ubiquitin–proteasome system [28]. The loss-of-function in VHL in this disease abolishes its intrinsic enzymatic activity as a ubiquitin E3 ligase, which in turn stabilizes HIF1α and promotes dimerization with constitutive HIF1β and translocation into nucleus, wherein HIF1α functions as the mast transcription factor to regulate diverse downstream gene and consequently contributes to the oncogenesis [29, 30]. Salama et al. demonstrated the stabilized HIF1α in VHL mutant clear cell renal cell carcinoma up-regulated sphingosine kinase-1, which in turn induced invasion in an autocrine manner and angiogenesis in a paracrine manner [31]. Razorenova et al. reported that the apoptosis repressor with a CARD domain (ARC) gene was a direct hypoxia-inducible factor 1 target gene and promoted survival and proliferation of VHL-deficient renal cancer cells [32]. Notably, more than 90% of the most common form of kidney cancer are associated with bi-allelic somatic mutation in VHL gene. Here we shed light on the previously unrecognized molecular mechanism underlying aberrant activation of hypoxia response in VHL-intact renal carcinoma and our data uncovered an alternative pathway through which hypoxia response was activated. Consistent with previous study that suggested miR-199a-5p negatively and post-transcriptionally regulated HIF1α in brain tissue of patients with intractable epilepsy [27], here we consolidated this regulatory axis in renal carcinoma which was further subjected to SNHG12 competition.

The anti-tumor properties of miR-199a-5p were increasingly recognized in range of human malignancies. For instance, Lian et al. disclosed the inhibitory effects of miR-199, miR-140 and miR-221 in Marek’s disease tumorigenesis [33]. Chen et al. demonstrated the epigenetically suppressed miR-199/miR-214 via PSMD10/TP53/DNMT1 self-regulatory network in testicular germ cell tumor [34]. The prognostic value of miR-199-5p along with the comprehensive microRNA expression profile has been proposed in colorectal cancer [35]. Troppan et al. further demonstrated that miR-199a and miR-497 were associated with better overall survival in diffuse large B cell lymphoma patients due to improved chemo-sensitivity [36]. The miR-199 has also been exploited by Callegari et al. in oncolytic adenovirus for cancer therapeutics [37]. The results from Amr et al. implicated the potential value of miR-21 and miR-199a in early diagnosis of hepatocellular carcinoma as well [38]. In support of the crucial anti-tumor function of miR-199a-5p, here we demonstrated that ectopic introduction of miR-199a-5p significantly suppressed the malignant behavior in renal carcinoma cells, which held great promise for the therapeutic purpose in this disease.

Noteworthily, the molecular mechanism underlying high-expression of SNHG12 in renal carcinoma was still to be elucidated in our future investigations. The previous study indicated that SNHG12 was subjected to the regulation by the transcription factor C-Myc, which was frequently over-activated in renal carcinoma [19]. Therefore, we hypothesized that the same scenario might operate in renal carcinoma, which definitely warranted further experimental validation.

In summary, here we identified the oncogenic property of SNHG12 via miR-199a-5p-HIF1α pathway, which contributed to our comprehensive understanding of the hypoxia response in VHL-proficient renal carcinoma.

## Conclusion

We characterize aberrant over-expression of SNHG12 in renal carcinoma, which competed with miR-199a-5p to positively regulate HIF1α. Our study unraveled a novel mechanism underlying hypoxic context in VHL-proficient renal cancer, which held potential promise for diagnostic and therapeutic exploitations.

## References

[1] Capitanio U, Montorsi F (2016). Renal cancer. Lancet.

[2] Choueiri TK, Pomerantz MM, Signoretti S (2013). Renal-cell carcinoma: a step closer to a new classification. Lancet Oncol.

[3] Siegel R, Ma J, Zou Z, Jemal A (2014). Cancer statistics, 2014. CA Cancer J Clin.

[4] Lopez-Beltran A, Cheng L, Vidal A, Scarpelli M, Kirkali Z, Blanca A, Montironi R (2013). Pathology of renal cell carcinoma: an update. Anal Quant Cytopathol Histpathol.

[5] Ljungberg B, Campbell SC, Choi HY, Jacqmin D, Lee JE, Weikert S, Kiemeney LA (2011). The epidemiology of renal cell carcinoma. Eur Urol.

[6] Davis CF, Ricketts CJ, Wang M, Yang L, Cherniack AD, Shen H, Buhay C, Kang H, Kim SC, Fahey CC (2014). The somatic genomic landscape of chromophobe renal cell carcinoma. Cancer Cell.

[7] Haddad AQ, Margulis V (2015). Tumour and patient factors in renal cell carcinoma-towards personalized therapy. Nat Rev Urol.

[8] Pal SK, Haas NB (2014). Adjuvant therapy for renal cell carcinoma: past, present, and future. Oncologist.

[9] Funakoshi T, Lee CH, Hsieh JJ (2014). A systematic review of predictive and prognostic biomarkers for VEGF-targeted therapy in renal cell carcinoma. Cancer Treat Rev.

[10] Thomas JS, Kabbinavar F (2015). Metastatic clear cell renal cell carcinoma: a review of current therapies and novel immunotherapies. Crit Rev Oncol Hematol.

[11] Perkel JM (2013). Visiting “noncodarnia”. Biotechniques.

[12] Mercer TR, Dinger ME, Mattick JS (2009). Long non-coding RNAs: insights into functions. Nat Rev Genet.

[13] Goodrich JA, Kugel JF (2006). Non-coding-RNA regulators of RNA polymerase II transcription. Nat Rev Mol Cell Biol.

[14] Engreitz JM, Haines JE, Perez EM, Munson G, Chen J, Kane M, McDonel PE, Guttman M, Lander ES (2016). Local regulation of gene expression by lncRNA promoters, transcription and splicing. Nature.

[15] Sanchez-Elsner T, Gou D, Kremmer E, Sauer F (2006). Noncoding RNAs of trithorax response elements recruit Drosophila Ash1 to Ultrabithorax. Science.

[16] Zhou S, Yu L, Xiong M, Dai G (2018). LncRNA SNHG12 promotes tumorigenesis and metastasis in osteosarcoma by upregulating Notch2 by sponging miR-195-5p. Biochem Biophys Res Commun.

[17] Zhang J, Yuan L, Zhang X, Hamblin MH, Zhu T, Meng F, Li Y, Chen YE, Yin KJ (2016). Altered long non-coding RNA transcriptomic profiles in brain microvascular endothelium after cerebral ischemia. Exp Neurol.

[18] Wang JZ, Xu CL, Wu H, Shen SJ (2017). LncRNA SNHG12 promotes cell growth and inhibits cell apoptosis in colorectal cancer cells. Braz J Med Biol Res.

[19] Wang O, Yang F, Liu Y, Lv L, Ma R, Chen C, Wang J, Tan Q, Cheng Y, Xia E (2017). C-MYC-induced upregulation of lncRNA SNHG12 regulates cell proliferation, apoptosis and migration in triple-negative breast cancer. Am J Transl Res.

[20] Zhu TG, Xiao X, Wei Q, Yue M, Zhang LX (2017). Revealing potential long non-coding RNA biomarkers in lung adenocarcinoma using long non-coding RNA-mediated competitive endogenous RNA network. Braz J Med Biol Res.

[21] Wang X, Qi G, Zhang J, Wu J, Zhou N, Li L, Ma J (2017). Knockdown of long noncoding RNA small nucleolar RNA host gene 12 inhibits cell growth and induces apoptosis by upregulating miR-138 in nonsmall cell lung cancer. DNA Cell Biol.

[22] Wang P, Chen D, Ma H, Li Y (2017). LncRNA SNHG12 contributes to multidrug resistance through activating the MAPK/Slug pathway by sponging miR-181a in non-small cell lung cancer. Oncotarget.

[23] Zhang H, Lu W (2018). LncRNA SNHG12 regulates gastric cancer progression by acting as a molecular sponge of miR320. Mol Med Rep.

[24] Tay Y, Rinn J, Pandolfi PP (2014). The multilayered complexity of ceRNA crosstalk and competition. Nature.

[25] Myszczyszyn A, Czarnecka AM, Matak D, Szymanski L, Lian F, Kornakiewicz A, Bartnik E, Kukwa W, Kieda C, Szczylik C (2015). The role of hypoxia and cancer stem cells in renal cell carcinoma pathogenesis. Stem Cell Rev.

[26] Jiang G, Zhou R, He X, Shi Z, Huang M, Yu J, Wang X (2016). Expression levels of microRNA-199 and hypoxia-inducible factor-1 alpha in brain tissue of patients with intractable epilepsy. Int J Neurosci.

[27] Lan T, Ma W, Hong Z, Wu L, Chen X, Yuan Y (2017). Long non-coding RNA small nucleolar RNA host gene 12 (SNHG12) promotes tumorigenesis and metastasis by targeting miR-199a/b-5p in hepatocellular carcinoma. J Exp Clin Cancer Res.

[28] Shen C, Kaelin WG (2013). The VHL/HIF axis in clear cell renal carcinoma. Semin Cancer Biol.

[29] Arjumand W, Sultana S (2012). Role of VHL gene mutation in human renal cell carcinoma. Tumour Biol.

[30] Schodel J, Grampp S, Maher ER, Moch H, Ratcliffe PJ, Russo P, Mole DR (2016). Hypoxia, hypoxia-inducible transcription factors, and renal cancer. Eur Urol.

[31] Salama MF, Carroll B, Adada M, Pulkoski-Gross M, Hannun YA, Obeid LM (2015). A novel role of sphingosine kinase-1 in the invasion and angiogenesis of VHL mutant clear cell renal cell carcinoma. FASEB J.

[32] Razorenova OV, Castellini L, Colavitti R, Edgington LE, Nicolau M, Huang X, Bedogni B, Mills EM, Bogyo M, Giaccia AJ (2014). The apoptosis repressor with a CARD domain (ARC) gene is a direct hypoxia-inducible factor 1 target gene and promotes survival and proliferation of VHL-deficient renal cancer cells. Mol Cell Biol.

[33] Lian L, Zhang D, Wang Q, Yang N, Qu L (2015). The inhibitory effects of gga-miR-199-3p, gga-miR-140-3p, and gga-miR-221-5p in Marek’s disease tumorigenesis. Poult Sci.

[34] Chen BF, Suen YK, Gu S, Li L, Chan WY (2014). A miR-199a/miR-214 self-regulatory network via PSMD10, TP53 and DNMT1 in testicular germ cell tumor. Sci Rep.

[35] Pecqueux M, Liebetrau I, Werft W, Dienemann H, Muley T, Pfannschmidt J, Mussle B, Rahbari N, Scholch S, Buchler MW (2016). A comprehensive MicroRNA expression profile of liver and lung metastases of colorectal cancer with their corresponding host tissue and its prognostic impact on survival. Int J Mol Sci.

[36] Troppan K, Wenzl K, Pichler M, Pursche B, Schwarzenbacher D, Feichtinger J, Thallinger GG, Beham-Schmid C, Neumeister P, Deutsch A (2015). miR-199a and miR-497 are associated with better overall survival due to increased chemosensitivity in diffuse large B-cell lymphoma patients. Int J Mol Sci.

[37] Callegari E, Elamin BK, D’Abundo L, Falzoni S, Donvito G, Moshiri F, Milazzo M, Altavilla G, Giacomelli L, Fornari F (2013). Anti-tumor activity of a miR-199-dependent oncolytic adenovirus. PLoS ONE.

[38] Amr KS, Ezzat WM, Elhosary YA, Hegazy AE, Fahim HH, Kamel RR (2016). The potential role of miRNAs 21 and 199-a in early diagnosis of hepatocellular carcinoma. Gene.

